# Methodological Limitations of CBCT-Derived Gray Values in Assessing Radiographic Attenuation Patterns After Peri-Implantitis Surgery: Secondary Analysis of a Prospective Clinical Cohort

**DOI:** 10.3390/jcm15114144

**Published:** 2026-05-27

**Authors:** Katarzyna Wieczorek, Grzegorz Hajduk, Michał Łobacz, Paulina Mertowska, Ewelina Grywalska, Sebastian Mertowski, Daya Masri

**Affiliations:** 1 Chair and Department of Oral Surgery, Medical University of Lublin, 20-093 Lublin, Poland; esso53@wp.pl (G.H.); michal.lobacz@umlub.pl (M.Ł.); 2 Department of Experimental Immunology, Medical University of Lublin, 20-093 Lublin, Poland; paulinamertowska@umlub.pl (P.M.); ewelina.grywalska@umlub.pl (E.G.); sebastian.mertowski@umlub.pl (S.M.); 3 Department of Oral and Maxillofacial Surgery, The Maurice and Gabriela Goldschleger School of Dental Medicine, Tel Aviv University, Tel Aviv 6997801, Israel; dr.dayamasri@gmail.com

**Keywords:** cone-beam computed tomography, gray values, HU-like values, radiographic attenuation, radiological reliability, measurement validity, peri-implantitis, peri-implant bone defects, methodological study, secondary analysis

## Abstract

**Objectives**: Cone-beam computed tomography (CBCT) is central to three-dimensional assessment in oral surgery and implant dentistry; however, CBCT-derived gray values expressed as HU-like units are not equivalent to true CT-derived Hounsfield Units (HU). This brief methodological secondary analysis evaluated the reliability and practical limitations of such values in assessing radiographic changes after peri-implantitis surgery. **Methods**: The analysis used the imaging protocol and group-level radiological data from a previously published prospective clinical cohort, conducted under the same protocol and ethical approval of the Institutional Ethics Committee of the Medical University of Lublin (KE-0254/248/11/2023; 23 November 2023). The source cohort included 57 patients treated after implant removal for severe peri-implantitis with small-particle dentin (n = 22), Bio-Oss (n = 15), or spontaneous healing without grafting (n = 20). CBCT scans were analyzed in OnDemand3D (version 1.0.11.1007) using manually selected square regions of interest (ROI; 30 × 30 pixels). No external phantom calibration, cross-device normalization, or formal intra-/inter-observer reproducibility assessment was available in the secondary dataset. **Results**: The previously reported mean study-site values were 779.62 ± 325.92 gray-value units for small-particle dentin, 910.51 ± 155.03 gray-value units for Bio-Oss, and 206.04 ± 174.21 gray-value units for controls. These findings are presented as protocol-dependent attenuation patterns, not as direct material rankings, bone-density thresholds, or proof of regeneration. Variability remained substantial, with study-site coefficients of variation of 41.8%, 17.0%, and 84.6%, respectively, and high adjacent-site variability. Interpretation was constrained by manual ROI placement, lack of calibration, absence of observer-agreement metrics, unequal follow-up timing, and CBCT sensitivity to scatter, beam hardening, field of view, reconstruction settings, and metal-related artifacts. **Conclusions**: CBCT-derived gray values may be useful as relative indicators of local radiographic attenuation change within a standardized protocol, but they should not be interpreted as absolute measures of bone density. Future regenerative oral surgery studies should combine standardized acquisition, explicit ROI methodology, repeated measurements, observer-agreement analysis, and complementary clinical, radiographic, or histological outcomes.

## 1. Introduction

Accurate assessment of peri-implant bone morphology and regenerative outcome is essential for treatment planning, follow-up, and interpretation of surgical success in oral implantology. CBCT has become the preferred three-dimensional imaging modality in many dentoalveolar indications because it offers high spatial resolution and clinically useful depiction of defect morphology at a radiation burden that is generally lower than that of conventional multislice CT [1,2,3,4,5].

In conventional medical CT, HU are standardized attenuation numbers defined relative to water and air. CBCT reconstruction, in contrast, is optimized primarily for dentomaxillofacial spatial depiction rather than absolute densitometry, and CBCT gray values are not automatically anchored to a universal physical scale. They vary according to device architecture, detector behavior, exposure parameters, field of view, voxel size, reconstruction algorithm, patient positioning, beam hardening, scatter, and the presence of highly attenuating structures such as metal restorations or implants [6,7,8,9,10]. Attempts to improve interpretability have included phantom-based calibration, device-specific conversion approaches, and stricter standardization of acquisition and ROI protocols [6,8,9,10,11,12]. Nevertheless, these approaches do not make CBCT gray values universally interchangeable with true CT-derived HU; therefore, CBCT-derived values should be regarded as HU-like or protocol-dependent attenuation values rather than true HU.

The literature on this issue is heterogeneous. Several studies have reported substantial correlations between CBCT-derived attenuation values and multislice CT, micro-CT, or subjective surgical bone quality assessment, suggesting that CBCT may provide clinically meaningful information under controlled conditions [6,7,8,9,12,13,14,15]. Conversely, other studies have shown that CBCT is reliable mainly for linear measurements rather than for true quantitative densitometry, and some authors have found poor agreement with histology or a lack of genuine HU calibration [10,11,16]. Repeatability may also vary according to region of interest, field of view, and software settings [9,12]. From a clinical perspective, overinterpreting CBCT-derived gray values may affect implant site evaluation, timing of re-entry or implant placement, graft monitoring, and claims regarding biomaterial performance. Recent clinical and radiographic literature also indicates that long-term regenerative stability, postoperative healing, and implant stability depend on multiple biological, procedural, and imaging-related variables rather than on a single attenuation metric [17,18,19].

Our group recently reported clinical and radiological outcomes of regenerative management after peri-implantitis surgery in a prospective cohort that compared small-particle dentin, Bio-Oss, and spontaneous healing [20]. In that study, CBCT-derived attenuation values were used as the principal radiological endpoint. The efficacy findings were clinically informative, but the protocol also highlighted important measurement issues: manual selection of a small ROI, unequal follow-up intervals between groups, large within-group dispersion, and potential artifact susceptibility in post-explantation sites.

The present study was therefore designed as a methodological secondary analysis of that cohort. The aim was not to re-evaluate biomaterial efficacy or to propose new universal densitometric thresholds, but to examine the interpretative limits of CBCT-derived gray values in this setting and to define how such values should be reported and qualified in future regenerative studies. The intended contribution is a clinically contextualized methodological caution and reporting framework rather than a claim that the general lack of CBCT HU calibration is a new finding. The null hypothesis was that CBCT-derived HU-like values can be interpreted as direct and absolute measures of bone density in post-surgical peri-implant sites. The alternative hypothesis was that these values behave primarily as relative, protocol-dependent indicators of radiographic attenuation rather than as true densitometric measurements.

## 2. Materials and Methods

### 2.1. Study Design

This manuscript presents a methodological secondary analysis of radiological data obtained in a previously reported prospective clinical cohort by Łobacz et al. [20]. The original cohort included 57 adults with severe peri-implantitis requiring implant explantation and defect management. Patients were allocated to three groups according to the material used clinically: small-particle dentin (n = 22), Bio-Oss (Geistlich Pharma AG, Wolhusen, Switzerland; n = 15), and spontaneous healing without a bone substitute (n = 20) [20]. The mean study-site attenuation values and paired adjacent-site values analyzed below are derived from that source cohort and are reinterpreted here for methodological purposes rather than presented as new primary clinical efficacy outcomes.

The clinical efficacy outcomes of this cohort have already been published [20]. The present paper addresses a different research question, namely, the measurement behavior and interpretative validity of CBCT-derived attenuation values in this model. No new patient recruitment, intervention, or additional radiological exposure was performed for the current analysis.

The original study was conducted in accordance with the Declaration of Helsinki and approved by the Institutional Ethics Committee of the Medical University of Lublin (KE-0254/248/11/2023; approval date: 23 November 2023). All participants provided informed consent [20].

### 2.2. Clinical and Surgical Context of the Source Cohort

In the source cohort, patients had severe peri-implantitis that required implant explantation followed by management of the post-explantation bone defect [20]. Because implants were removed, implant-surface decontamination was not a procedural endpoint for the present radiological analysis. The clinically relevant surgical sequence consisted of implant removal, debridement of the defect, removal of visible inflammatory or granulation tissue, and subsequent defect management according to group allocation: small-particle dentin grafting, Bio-Oss (Geistlich Pharma AG, Wolhusen, Switzerland) grafting, or spontaneous healing without grafting [20]. The practical surgical endpoint before defect management was a clinically clean post-explantation defect with removal of visible pathological soft tissue. The present manuscript does not reanalyze surgical efficacy; instead, it addresses how the resulting CBCT gray values should be interpreted methodologically.

This surgical context is important because residual inflammatory tissue, sclerotic bone, irregular defect morphology, residual graft particles, and local anatomical heterogeneity may all influence radiographic attenuation. Therefore, even with a consistent operative protocol, CBCT-derived gray values cannot isolate biological regeneration from measurement variability unless the imaging protocol, ROI selection, timing, and validation methods are also standardized.

### 2.3. Imaging Protocol and ROI Selection

In the source cohort, baseline and follow-up CBCT scans were analyzed with the OnDemand3D App (version 1.0.11.1007; Cybermed Inc., Seoul, Republic of Korea). All numerical analyses in the present paper were therefore performed within the same software framework as the source cohort. The region of interest was selected manually using the software polygon/ROI tools, and square areas measuring 30 × 30 pixels were used for the measurements. Within each ROI, the software provided the minimum, maximum, mean, and standard deviation values for attenuation [20,21]. This manual ROI approach is clinically practical but operator dependent; therefore, future quantitative studies should report the reconstructed plane, ROI dimensions, anatomical landmarks, observer training, and repeatability procedure in sufficient detail.

Because the present work is a secondary analysis of previously published group-level data, it could not introduce new scanner calibration or retrospective raw-data normalization. Complete device-level acquisition parameters required for independent densitometric validation, including full scanner settings, field of view, voxel size, reconstruction parameters, and artifact-reduction settings, were not available for reanalysis in the current dataset. Consequently, the numerical values reported here should be interpreted as protocol-bound CBCT-derived attenuation values rather than transferable HU thresholds.

No external phantom calibration was performed in the source protocol, and the available secondary dataset did not contain a reproducibility log documenting repeated ROI placement by calibrated independent observers. Therefore, formal intra-observer or inter-observer agreement metrics, such as intra-class correlation coefficients, could not be calculated. This absence of calibration and reproducibility testing is treated as a central methodological limitation of the present analysis rather than as evidence that the measurements are reproducible.

Follow-up timing was not identical across groups. In the small-particle dentin group, evaluation was performed after 8 weeks, in line with the protocol used for the dentin-derived graft material [20,22]. In the Bio-Oss and control groups, radiological assessment was performed after 12 weeks, corresponding to a later stage of socket maturation and defect healing [20,23].

In addition to the treated site, an adjacent site was analyzed as an internal local comparator. This approach offered a useful intra-patient reference, but it did not eliminate the influence of local heterogeneity in trabecular architecture, cortical thickness, residual defects, or reconstruction artifacts.

### 2.4. Methodological Endpoints

The primary methodological endpoints of the present analysis were: (1) dispersion of CBCT-derived values within each group, expressed as standard deviation and coefficient of variation (CV%); (2) local contrast between study-site and adjacent-site values, expressed as absolute difference and study-to-adjacent ratio; and (3) protocol-level threats to measurement validity, including ROI dependence, artifact burden, absence of external calibration, unmeasured observer reproducibility, non-equivalent postoperative time points, and the absence of a reference validation method.

Because the present analysis was focused on measurement interpretation, emphasis was placed on whether the reported values could plausibly support statements about absolute bone density. Direct intergroup claims about biological efficacy were deliberately avoided because the source cohort used non-equivalent follow-up windows, and the present secondary analysis did not include raw-data reanalysis or reference validation.

### 2.5. Statistical Analysis

The original study used Shapiro–Wilk testing to assess normality, Student’s t-test for dependent samples for paired comparisons, and ANOVA with Tukey post hoc testing for group comparisons, with statistical significance set at *p* < 0.05 [20]. In the present brief methodological report, those source-cohort inferential tests are not used to establish material superiority or validated densitometric thresholds. The current analysis is descriptive, exploratory, and focused on dispersion, local contrast, and threats to validity. Because the present reanalysis was based on published group-level summaries rather than individual raw measurements, uncertainty propagation from the original ROI-level data could not be modeled, and no reproducibility statistics such as intra-class correlation coefficients could be calculated.

For the present methodological paper, additional descriptive dispersion metrics were derived from the published group-level summary data. The coefficient of variation was calculated as the standard deviation divided by the mean value multiplied by 100. Study-to-adjacent ratios were also calculated to quantify the magnitude of local contrast relative to the internal reference site. Because repeated raw measurements were not available for this secondary analysis, intra-class correlation coefficients, Bland–Altman limits, or other observer-agreement statistics could not be calculated. Formal propagation of measurement uncertainty from pixel-level or repeated ROI-level data was also not possible; uncertainty is therefore discussed qualitatively through dispersion metrics and protocol-level threats to validity.

## 3. Results

### 3.1. Magnitude and Dispersion of CBCT-Derived Values

Table 1 summarizes the descriptive source-cohort CBCT-derived attenuation values and derived dispersion metrics. Study-site values were higher in the two grafted groups and lower in the control group. In the present methodological analysis, these values are reported to illustrate the behavior and dispersion of CBCT-derived gray values. They should not be interpreted as a direct efficacy ranking of the graft materials or as comparable absolute density endpoints, because postoperative imaging was performed at different time points across groups.

Within-protocol paired local contrasts showed higher study-site attenuation than adjacent-site attenuation in both grafted groups, whereas the control group showed a lower study-site than adjacent-site value. These paired local contrasts are presented as descriptive attenuation patterns and are not used here to validate absolute bone density or histological regeneration [20].

From a methodological perspective, dispersion was the principal result. Study-site CVs ranged from 17.0% in Bio-Oss to 84.6% in controls, while adjacent-site variability also remained considerable. This spread indicates that the numerical signal may be influenced by measurement instability and protocol effects in addition to biological heterogeneity.

**Figure 1:**
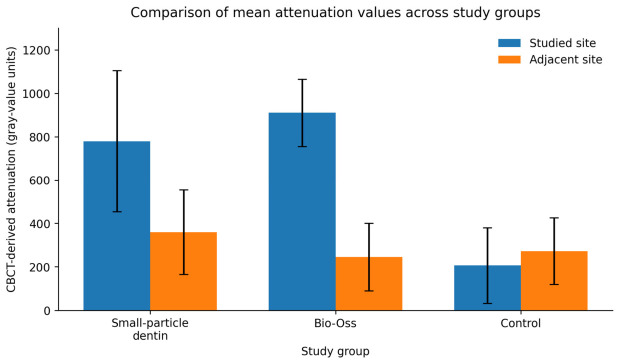
Descriptive comparison of mean CBCT-derived attenuation values at the studied and adjacent sites across the three groups. Error bars indicate SD. Because postoperative imaging intervals differed between groups, the figure should not be interpreted as a direct intergroup efficacy comparison.

The study-to-adjacent ratios further illustrated local contrast but also protocol dependence: ratios were above 1.0 in the grafted groups and below 1.0 in controls. Although these findings support a directional signal of local radiographic attenuation change, they do not by themselves validate the use of the reported values as absolute bone density estimates or direct indicators of tissue regeneration. Figure 1 provides a descriptive visual display only and should not be interpreted as an inferential comparison of regenerative efficacy between groups.

### 3.2. Protocol Features That Limited Quantitative Interpretation

The imaging protocol contained several features that constrained direct densitometric interpretation. First, the ROI was selected manually and was limited to a square of 30 × 30 pixels. In a healing post-explantation site, even a small change in ROI placement may capture different proportions of cortical bone, trabecular bone, marrow spaces, graft particles, or voids, thereby altering the mean attenuation.

Second, the postoperative observation windows were not equivalent across groups, with dentin assessed after 8 weeks and Bio-Oss and controls after 12 weeks. Because mineralization, graft remodeling, and radiographic attenuation evolve over time, this design precludes definitive intergroup interpretation of attenuation differences as material-specific density effects.

Third, the protocol did not include phantom-based calibration or cross-device normalization. Fourth, no formal repeated-measurement or observer-agreement dataset was available. Therefore, the reported values should be interpreted within the same acquisition and analysis setting only and should not be generalized as transferable HU thresholds.

## 4. Discussion

The present paper re-examined a prospective peri-implantitis cohort from a methodological angle and showed that the interpretation of CBCT-derived gray values requires substantial caution. The manuscript does not claim that the absence of universal HU calibration in CBCT is a new discovery. Rather, its contribution is to demonstrate how this known limitation affects interpretation in a real post-explantation peri-implantitis cohort and to translate the problem into practical reporting and quality-control recommendations. The original cohort demonstrated clinically relevant local radiographic differences between grafted and ungrafted defects [20]. Nevertheless, the same dataset also showed wide dispersion, protocol dependence, and several structural threats to validity that limit the use of these values as absolute measures of bone density or as direct radiological proof of tissue regeneration.

The first key point is conceptual. In medical CT, HU are physically anchored to calibrated attenuation coefficients. In CBCT, gray values may correlate with density under selected conditions, but they are not inherently equivalent to HU. A systematic review and meta-analysis by Selvaraj et al. [8] summarized the heterogeneity of correlations between CBCT gray values and CT HU, while other studies demonstrated that ROI choice, field of view, and software settings influence measured values [10,11,12]. These findings support the central conclusion that CBCT-derived gray values should be treated as protocol-dependent attenuation indicators, not as universal densitometric units.

The second key issue is ROI dependence. The use of a manually selected 30 × 30 pixel square may be practical in routine software, but it is highly sensitive to operator decisions. In a healing post-explantation socket or peri-implant defect, minor shifts in ROI position may alter the fraction of residual graft particles, immature woven bone, marrow spaces, and cortical margins included in the measurement. This issue is especially relevant when dispersion is already large, as seen in the small-particle dentin and control groups. Under such conditions, a single mean value can give a misleading impression of quantitative precision. Because formal intra- and inter-observer reproducibility was not available in the secondary dataset, this source of measurement uncertainty could not be separated from biological variability.

The third issue is artifact burden. CBCT around implant sites is particularly vulnerable to scatter and beam hardening because of metallic restorations, implant remnants, dense graft particles, cortical boundaries, and adjacent prosthetic or endodontic materials. Scatter can introduce non-uniform gray-value shifts and reduce local contrast, whereas beam hardening may produce streaks, cupping effects, or artificial bright and dark bands near highly attenuating structures. These phenomena may either inflate or depress local attenuation values depending on geometry, field of view, reconstruction settings, and the position of the ROI. Objective mitigation in future studies should include reporting whether metal artifact reduction was used, excluding or separately analyzing ROIs located within obvious artifact zones, documenting artifact burden, and validating gray values with calibration phantoms or reference imaging when quantitative interpretation is intended. In the present secondary dataset, such artifact scoring and correction were not available; therefore, variability in this setting may reflect image physics as much as tissue mineralization.

To make these limitations more transparent and clinically usable, Table 2 summarizes the principal sources of uncertainty when CBCT-derived gray values are interpreted as calibrated densitometric values in regenerative oral surgery studies and lists practical mitigation strategies.

Another important limitation concerns follow-up timing. In the analyzed protocol, the small-particle dentin group was assessed after 8 weeks, whereas the Bio-Oss and control groups were assessed after 12 weeks [20,22,23]. This design was clinically understandable because the healing kinetics of autogenous dentin and xenograft-based socket preservation may differ. However, from a measurement standpoint, non-equivalent observation windows complicate intergroup interpretation. A higher or lower attenuation value at a given time point may reflect the biological stage of remodeling, residual graft persistence, or maturation pattern rather than a directly comparable density endpoint. For this reason, the revised Results section presents between-group values descriptively and avoids using them as direct comparative efficacy evidence.

The clinical implication is not that CBCT should be abandoned. On the contrary, CBCT remains valuable for assessing three-dimensional defect morphology, ridge dimensions, cortical integrity, proximity to anatomical structures, and the spatial pattern of healing [3,4,5,24]. In the source cohort, CBCT was used because the post-explantation defects were three-dimensional and the clinical question concerned spatial defect healing. However, clinical decisions should not be based on a single CBCT gray-value measurement. A more defensible approach is to integrate three-dimensional morphology, standardized radiographic follow-up, clinical healing, implant stability when relevant, and long-term stability of the regenerated site [17,18,19].

Standardized intraoral radiographs with reproducible geometry remain highly relevant for longitudinal monitoring of peri-implant marginal bone levels and should not be replaced by CBCT-derived gray-value measurements when a two-dimensional marginal bone-level question is sufficient [16,25,26]. The role of CBCT should be distinguished from that of standardized intraoral radiography: CBCT may be justified for three-dimensional defect configuration and treatment planning, whereas CBCT gray values should not be used as absolute HU-based density thresholds.

For future studies, several practical improvements should be considered. Full reporting of CBCT acquisition parameters, including scanner model, exposure settings, field of view, voxel size, reconstruction parameters, and artifact-reduction settings, should be standard. Phantom-based calibration would provide an external reference and permit at least partial normalization of gray values. Repeated measurements by independent observers would allow calculation of intra-class correlation coefficients, Bland–Altman limits, and within-ROI coefficients of variation. Larger or volumetric ROIs could reduce sensitivity to local pixel-level variation, although they would need carefully standardized anatomical boundaries. Finally, radiological endpoints should be interpreted together with standardized intraoral radiographs, clinical healing, implant stability when relevant, histomorphometry when available, or, at a minimum, longitudinal within-subject trends and long-term graft stability outcomes [17,18,19].

A practical minimum ROI workflow for future studies would be: (1) define anatomical landmarks and ROI dimensions before analysis; (2) place the ROI in the same reconstructed plane and at the same distance from defect boundaries whenever possible; (3) perform at least three repeated ROI placements per site; (4) use at least two calibrated observers; (5) report mean, SD, CV, ICC, and agreement limits; and (6) predefine quality-control flags for high dispersion. Because validated universal CV thresholds for CBCT gray-value reliability are not available, any threshold should be treated as a study-specific quality-control flag rather than a clinical truth criterion. As a pragmatic reporting convention, CV values above 40% should prompt caution, sensitivity analysis, or avoidance of threshold-based interpretation, while lower CV values should still require calibration and observer-agreement confirmation before clinical thresholding.

The present study has limitations. It was a single-center secondary analysis of one prospective cohort. The methodological re-interpretation relied on the reported imaging protocol and published radiological summary data, and complete raw acquisition parameters, phantom data, repeated observer measurements, artifact scoring, and histological validation were not available for the present reanalysis. Therefore, the paper cannot determine what percentage of a reported HU-like value corresponds to actual mineral density and cannot replace a dedicated validation study comparing CBCT with phantom-calibrated CT, micro-CT, or histology. In addition, because only aggregated summary data were available, uncertainty from the original ROI-level measurements could not be propagated into new inferential models. No material-specific HU or gray-value thresholds for differential diagnosis of regeneration are proposed. The added contextual literature on graft stability, healing outcomes, and implant stability helps frame the clinical relevance of the problem but does not provide direct validation of the present CBCT-derived gray values. Nevertheless, the analysis addresses a common but insufficiently discussed issue in dental research: the tendency to report CBCT gray values as though they were universal HU, without equivalent calibration or agreement testing.

Taken together, the evidence suggests that the null hypothesis should be rejected. In post-surgical peri-implant defects, CBCT-derived gray values should not be regarded as direct and absolute surrogates of bone density. Their value lies in supporting structured, within-protocol, preferably within-patient comparisons under rigorously standardized conditions, not in providing transferable densitometric truth or validated regenerate thresholds.

## 5. Conclusions

Within the limitations of this methodological secondary analysis, the null hypothesis that CBCT-derived gray-value attenuation measurements can be interpreted as direct and absolute measures of bone density in post-surgical peri-implant sites should be rejected. The alternative hypothesis was supported: CBCT-derived gray values may detect directional local radiographic attenuation differences under a standardized protocol, but they do not behave as robust absolute densitometric measurements or direct indicators of tissue regeneration.

Large dispersion, manual ROI dependence, lack of phantom calibration, artifact susceptibility, absence of observer-agreement data, lack of reference validation, and different postoperative observation windows limit quantitative interpretation of the reported values as true HU and preclude the formulation of transferable diagnostic thresholds.

For regenerative oral surgery studies, CBCT-derived gray-value metrics should be reported as relative, protocol-specific indicators and should be accompanied by explicit acquisition details, reproducible ROI methodology, repeated measurements, observer-agreement analysis, artifact assessment, and complementary clinical, standardized radiographic, or histological outcomes. Direct intergroup efficacy claims should be avoided when follow-up intervals are not equivalent.

## Figures and Tables

**Table 1:** Summary of CBCT-derived gray values expressed as HU-like attenuation units and derived dispersion metrics.

Group	Study Site Mean (Gray-Value Units)	Study Site SD	Study Site CV (%)	Adjacent Site Mean (Gray-Value Units)	Adjacent Site SD	Adjacent Site CV (%)	Study/Adjacent Ratio
Small-particle dentin	779.62	325.92	41.8	359.75	195.10	54.2	2.17
Bio-Oss	910.51	155.03	17.0	245.23	155.29	63.3	3.71
Control	206.04	174.21	84.6	272.19	153.06	56.2	0.76

CV, coefficient of variation. Ratios above 1.0 indicate higher attenuation at the treated site than at the adjacent reference site.

**Table 2:** Main sources of uncertainty when CBCT-derived gray values are interpreted as calibrated densitometric values in regenerative oral surgery studies.

Domain	Specific Issue	Potential Effect on Measured Value	Recommended Mitigation
Acquisition and calibration	Absence of phantom calibration or external density reference	Gray values cannot be assumed to represent absolute HU across devices or even across protocols	Use calibration phantoms, fixed settings, and device-specific validation
ROI methodology	Manual 30 × 30 pixel ROI placement	Operator dependence; different proportions of graft, cortical bone, trabecular bone, and voids may be sampled	Use predefined landmarks, repeated placements, calibrated observers, ICC/agreement limits, and CV flags
Metal and scatter artifacts	Residual metallic structures, implant-related beam hardening, and scatter	Artificial inflation or depression of local gray values near the region of interest	Report MAR settings, score artifacts, avoid obvious artifact zones, and validate quantitatively with reference methods
Reconstruction settings	Dependence on field of view, voxel size, and reconstruction algorithm	Limited comparability between scans or studies	Lock acquisition parameters and report them in full
Timing of assessment	Different postoperative intervals between study groups	Confounding by healing stage rather than by material characteristics	Use identical follow-up windows or longitudinal mixed-effects analysis
Biological heterogeneity	Local variation in maxillary/mandibular trabeculation and cortical thickness	Large intrinsic spread even within adjacent regions	Use paired intra-patient comparisons and supplement with clinical or histological endpoints
Reference validation	No micro-CT, histology, or phantom-calibrated CT reference	Cannot determine the percentage correspondence between gray values and actual mineral density	Do not define universal thresholds without external validation
Clinical imaging context	Using CBCT gray values as a substitute for standardized marginal bone-level radiography	May overstate the role of CBCT for routine longitudinal monitoring	Use intraoral radiographs for longitudinal marginal bone-level follow-up when appropriate; reserve CBCT for 3D defect morphology and planning
Quality-control dispersion flag	High CV despite repeated ROI measurements	Risk of false precision and threshold-based overinterpretation	Use CV thresholds only as study-specific warnings; in the absence of external validation, CV >40% should prompt caution, sensitivity analysis, or avoidance of threshold-based interpretation

The table is intended as a practical reporting framework for future clinical studies rather than as a ranking of error magnitude.

## Data Availability

Research data resulting from the implementation of this study are available upon written request from the corresponding author.

## Abbreviations

The following abbreviations are used in this manuscript:

ANOVA	analysis of variance
CBCT	cone-beam computed tomography
CT	computed tomography
CV	coefficient of variation
HU	Hounsfield units
ICC	intra-class correlation coefficient
IO	intraoral
ROI	region of interest
SD	standard deviation
